# Development of an opportunistic diagnostic prediction algorithm for osteoporosis and fragility fracture risk estimates from forearm radiographs (The OFFER1 Study)

**DOI:** 10.1093/jbmrpl/ziae020

**Published:** 2024-03-15

**Authors:** Robert Meertens, Ben Lopez, Ben Crone, Mike Gundry, Emma Metcalfe-Smith, Warren Gibbard, Thomas Jubb, Fay Manning, Paul Scott, Richard McWilliam

**Affiliations:** University of Exeter, Medical Imaging Exeter, EX1 2LU, United Kingdom; Ibex Innovations Ltd., Sedgefield, TS21 3FD, United Kingdom; Ibex Innovations Ltd., Sedgefield, TS21 3FD, United Kingdom; University of Exeter, Medical Imaging Exeter, EX1 2LU, United Kingdom; Ibex Innovations Ltd., Sedgefield, TS21 3FD, United Kingdom; Ibex Innovations Ltd., Sedgefield, TS21 3FD, United Kingdom; Ibex Innovations Ltd., Sedgefield, TS21 3FD, United Kingdom; University of Exeter, Medical Imaging Exeter, EX1 2LU, United Kingdom; Ibex Innovations Ltd., Sedgefield, TS21 3FD, United Kingdom; Ibex Innovations Ltd., Sedgefield, TS21 3FD, United Kingdom

**Keywords:** radiology, screening, fracture risk assessment, osteoporosis, DXA

## Abstract

Osteoporosis and associated fractures are an increasingly prevalent concern with an ageing population. This study reports testing of IBEX Bone Health (IBEX BH) software, applied following acquisition of forearm radiographs. IBEX Bone Health analyses the radiograph to measure areal bone mineral density (aBMD) at the examination site. A non-randomized cross-sectional study design was performed involving 261 (254 after exclusions) participants (112/142 m/f; mean age 70.8 years (SD+/–9.0); 53 with osteoporosis). They underwent posterior–anterior distal forearm radiographs; dual X-ray absorptiometry (DXA) of the wrists, hips, and lumbar spine; and questionnaires exploring clinical risk factors. IBEX Bone Health automatically identifies regions of interest (ROI) at the ultra-distal (UD) and distal third (TD) regions of the radius. Analysis investigated area under the receiver operating characteristics curve performance of IBEX BH for prediction of (i) osteoporosis (based on clinical reporting of the hip and spine DXA) and (ii) treatment recommendations by Fracture Risk Assessment Tool (FRAX) inclusive of neck of femur (NoF) areal bone mineral density (aBMD) results following National Osteoporosis Guideline Group (NOGG) guidelines. Area under the receiver operating characteristics curve for osteoporosis prediction at the UD and TD ROIs were 0.86 (99% confidence interval (CI) [0.80, 0.91]) and 0.81 (99% CI [0.75, 0.88]), respectively. Area under the receiver operating characteristics curve for treatment recommendation using FRAX inclusive of NoF aBMD at the UD and TD ROIs were 0.95 (99% CI [0.91, 1.00]) and 0.97 (99% CI [0.93,1.00]), respectively. With a matched sensitivity to FRAX (without NoF aBMD) 0.93 (99% CI [0.78, 0.99]), IBEX BH predicted at the UD and TD ROIs recommended treatment outcomes by NOGG guidelines using FRAX (with NoF aBMD) with specificity 0.89 (99% CI 0.83, 0.94]) and 0.93 (99% CI [0.87, 0.97]), respectively. This is compared with 0.60 (99% CI [0.51, 0.69]) for FRAX (without NoF aBMD). Results demonstrate the potential clinical utility of IBEX BH as an opportunistic screening tool.

## Introduction

In the presence of an ageing population, osteoporosis continues to emerge as an increasingly prevalent and debilitating disease. A recent meta-analysis reported an alarming worldwide prevalence of 18.3% (95% confidence interval [CI] 16.2–20.7)^1^ based on criteria defined by the World Health Organisation as an areal Bone Mineral Density (aBMD) 2.5 standard deviations (SDs) or lower than the mean value of a young, sex-matched population.^2^ Along with an alarmingly high prevalence, there is also widespread underdiagnosis that contributes to a large treatment gap (For the UK, there is a reported 66% treatment gap in women^3^).

Current treatment decision making is based on assessment of clinical risk factors (CRFs) and the current clinical standard for densitometry, dual X-ray absorptiometry (DXA). In the UK, National Osteoporosis Guideline Group (NOGG) guidance is often implemented, which recommends use of the validated clinical tool Fracture Risk Assessment Tool (FRAX). A FRAX assessment can be made based on CRFs alone, as the typical first step to determine whether referral for further investigation and DXA is warranted or whether the patient should be put directly onto treatment. Fracture Risk Assessment Tool estimates the 10-year percentage probability of hip fracture and major osteoporotic fracture to guide decision making for further referral and/or treatment. For example, a 10-year percentage probability of major osteoporotic fracture greater or equal to 10% is the “refer” threshold in those over 70, with an “intervention” threshold of 20% (i.e. medical treatment and/or specialist referral). Fracture Risk Assessment Tool can be recalculated with improved predictive power with the addition of a DXA measure of aBMD at the Neck of Femur (NoF).^4^^,^^5^

Associated fragility fractures, most commonly at the spine, hip, and wrists, ultimately lead to increased morbidity and mortality with an estimated 3.5 million new fragility fractures in Europe annually.^6^ The current allocation of resource in supporting those with osteoporosis is predominately reactionary, with 95% of the estimated annual €37billion economic burden dedicated to the treatment of fragility fracture and long-term fracture care rather than prevention.^6^ There is support for a refocusing of resources towards earlier identification as a priority for better patient outcomes, with an earlier initiation of preventative treatment prior to initial fragility fracture shown to have economic benefits for healthcare systems.^7^^,^^8^ Lifestyle and medical interventions have been demonstrated to reduce fragility fracture occurrence and preventative care is relatively inexpensive in comparison with treatment of primary fragility fractures, and by extension subsequent secondary fractures.^9^^,^^10^

However, DXA provision is low (7.5 units per million^11^) and variable across the UK (only 4 Fracture Liaison Services (FLSs) reported performing DXA within 90 days of fracture for 80% of cases^12^), especially since the COVID-19 pandemic. Furthermore, even if provision allowed, DXA requires proactive referral with a separate appointment for the patient based on CRFs and FRAX. The burden of identifying patients through CRFs and the cost of the additional scan – both to the health service and the patient’s time – hampers the potential for DXA and FRAX to close the treatment gap. A way to mitigate these problems would be to screen patients to identify those at high risk of osteoporosis, thus reducing the number of superfluous scans (i.e. those that show normal aBMD). Fracture liaison services are a way focusing the cohort on higher risk individuals since a fracture is a strong CRF for a future fracture with a Risk Ratio (RR) of 1.86 (95% CI [1.75, 1.98]).^13^ However, FLS will only pick up patients after they have experienced a fracture event so miss an opportunity to reduce (i) the cost of treating the primary fracture and (ii) the discomfort of the patient.

Numerous medical devices are available for the screening of bone health to facilitate earlier diagnosis of osteoporosis.^14^ However, these are still reliant on proactive referral based on recognition of CRFs. Further, evidence demonstrates that there is inconsistency in their application, despite established clinical guidance.^15^^,^^16^

A solution may lie in the emergence of medical imaging-based software applications that opportunistically screen for poor bone health as an incidental finding rather than a referral pathway.^17–22^ These approaches use imaging taken for any other reason to identify patients at high risk of osteoporosis without the need for an additional appointment, and with minimal impact on the acquisition and reporting process. However, existing software predominately rely on radiometric measurements of bone properties such as cortical thickness and textural analysis, which relies on the correlation to aBMD rather than measuring the actual biomarker.^17^^,^^18^^,^^20–22^

The IBEX Bone Health (IBEX BH) software provides an automated measurement of aBMD at the ultra-distal (UD) and distal third (TD) regions of interest (ROI).^19^ This quantitative approach solves the same fundamental problem as DXA measuring the same biomarker. Although the initial release of IBEX BH will only be used on wrist radiographs, additional trials are planned to extend IBEX BH to ankle, knee, and pelvis radiographs. The technique is not reliant upon large datasets of paired digital radiographs (DRs) and DXA scans to develop and it can therefore be extended to other body parts. The wrist was selected as it is a common imaging site in cases of arthritis and for suspected fragility fracture.

Although pelvis and lumbar sites are used for treatment decisions, wrist fractures are more prevalent in younger age brackets and are twice as likely in perimenopause than at other anatomical sites. Wrist fractures present a significant risk factor for subsequent fracture^23^ and the RR of fracture increases by 1.4 (95% CI [1.3, 1.6]) for every one SD decrease in aBMD at the distal radius. This is not statistically significantly lower than the equivalent measure for either the NoF 1.6 (95% CI [1.4, 1.8]) or the Lumbar Spine (LS) 1.5 (95% CI [1.4, 1.7]).^24^ Opportunistic screening at the wrist could identify a cohort of patients whose risk of fracture is equivalent to those patients that have already had a fracture and are not captured by FLSs. Although the adoption of wrist DXA is not as widespread as pelvis or spine for making treatment decisions, wrist radiographs are common (1 021 775 in 2019^25^) and predictive of fracture risk.

This paper explores the clinical utility of IBEX BH as an opportunistic screening tool within the current treatment decision framework; namely, its ability to identify patients with osteoporosis, and to predict the treatment recommendation from FRAX (inclusive of NoF aBMD).

## Materials and methods

The study received ethical approval from the United Kingdom (UK) Health Research Authority (Ref: 21/LO/0772) as well as institutional IRB approval. The study design was aligned with ethical principles outlined in the Declaration of Helsinki, including informed consent of all participants. A non-randomized cross-sectional study design was performed and reported with guidance from STROBE (Strengthening the reporting of observational studies in epidemiology. Data collection for each participant was performed in one appointment and included DXA scans of both wrists, both hips and the lumbar spine, bilateral PA wrist radiographs, and the FRAX questionnaire.

### Participants

**Figure 1:**
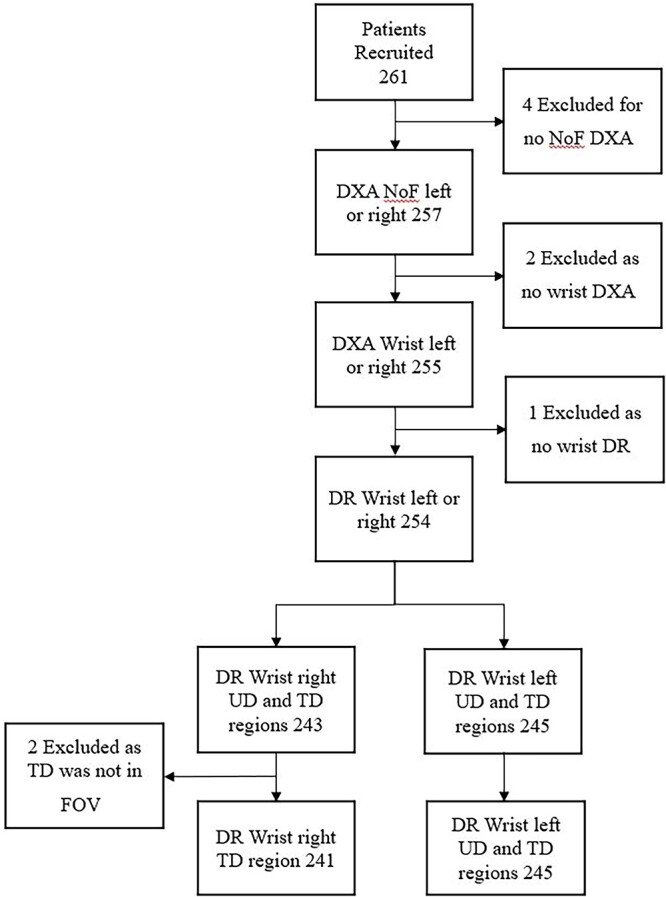
Participants flow chart showing recruitment and exclusions (neck of femur (NoF), ultra distal (UD), distal-third (TD), dual energy X-ray absorptiometry (DXA), and field of view (FOV)).

Non-randomized volunteer recruitment was performed with the target population participants over 50 years of age between February and October 2022 using primarily word of mouth recruitment including established participant networks at the University. Exclusion criteria included inability to consent, pregnancy, and/or previous bilateral fractures or surgical implantation at the distal radius and/or hip. Of the 261 participants recruited, 254 contributed data, culminating in the inclusion of (i) 488 wrists, (ii) 236 LSs, and (iii) hip DXA data from 492 hips. Therefore, a total of 254 participants had completed FRAX assessment inclusive with NoF aBMD data and at least one wrist DR and matching wrist DXA and were included in the statistical analysis. For 2 DR images the TD region was obscured by the collimator, so those ROIs were removed from the relevant analysis. A flow chart illustrating the exclusions is given in Figure 1.

Of the 261 participants, 25 (9.84%) were already on medical treatment for osteoporosis or had been prescribed treatment and had stopped taking it. Of these 25, (i) 16 (64%) had a T-score $\le$ –2.5 at either the NoF or the LS and (ii) 13 (52%) were recommended treatment by the NOGG guidelines on FRAX (with NoF aBMD).

### IBEX bone health

IBEX BH uses an inverse problem-solving approach to iteratively generate an anthropomorphic model of the anatomy being imaged. The underlying technology has been outlined in extensive detail previously.^26^^,^^27^ Each instance of the anatomy is imaged within a virtual X-ray system, using a simulation built using GEANT4.^28^ This provides a simulated version of the patient’s wrist radiograph. The difference relative to the actual radiograph is reduced through iterations of the anthropomorphic model, until a model of the anatomy is found that complies with the actual radiograph obtained within underlying errors in the system. The virtual X-ray system requires knowledge of the imaging geometry and exposure parameters used, as well as a statistical model encoding possible morphologies of the body part being examined. In contrast to DXA methods, which are the current mainstay of bone density imaging,^27^ this approach offers the advantage of alignment with standard DR imaging protocols.

aBMD and consequently T-score values can be inferred from the resultant anthropomorphic model by interrogating a specific region of interest (ROI) to assess the amount of bone present.^29^ T-scores derived at the examination site can then be used to calculate a probability of osteoporosis at other sites using multi-variate logistic regression analysis. A probability of being recommended treatment based on fragility fracture risk and NOGG guidelines can also be calculated using multi-variate logistic regression analysis by incorporating participant demographics and the CRFs present in FRAX (but only age and sex was used in this study).

**Figure 2:**
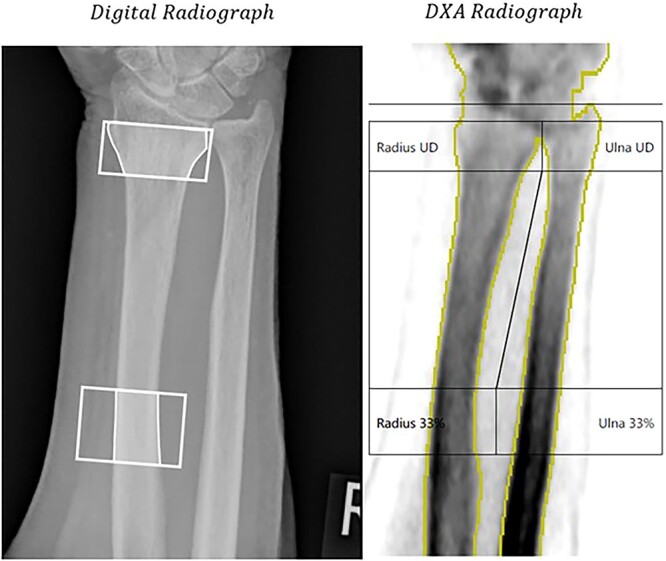
Left: Digital radiograph with ROIs shaded that are automatically produced using IBEX Bone Health. Right: Dual energy X-ray absorptiometry radiograph with ROIs after adjustment by reporting radiographer.

IBEX BH is fully automated, incorporating semantic segmentation of bone regions within the radiograph based on a convolutional neural network trained with 1158 hand-labelled radiographs. The architecture for this segmentation algorithm are given in Bullock et al.^30^, which reports the development of the network with a smaller training set. Placement of ROI at the ultra-distal (UD) and distal third (TD) regions of the radius are automated by a region-based instance segmentation algorithm trained on 1622 hand labelled radiographs utilizing the styloid process of the radius as a key reference point. Details of the training and validation of the ROI algorithm are given in Section 2 of the supplementary materials. Figure 2 shows a DR image from the study with the UD and TD ROIs found by IBEX BH overlayed. No data from participants enrolled in this study or taken on the X-ray device used in this study were included in the training of these algorithms.

### Data collection

Participants underwent bilateral posterior–anterior distal forearm radiographs with a fixed collimation field of 24 x 12 cm and a source to image distance of 115 cm on an AGFA DR100s mobile digital radiography system. Exposure factors were fixed to 60 kVp and 2 mAs.

Participants were surveyed by researchers using the FRAX^31^ questionnaire. Outputs of estimated 10-year major fragility fracture risk and hip fracture risk were recorded. This was repeated for FRAX with and without the NoF aBMD result collected during the study.

Participants underwent DXA scans of both wrists (UD and TD ROIs), hips (NoF and total hip), and LS (L1-L4) using clinically accepted scan protocols on a GE Lunar Prodigy Advanced DXA system. Forearm length was measured by tape measure as the distance from the tip of the ulnar styloid to the olecranon. T-scores were based on the GE default USA reference database, predominately derived from the National Health and Nutrition Examination Survey (NHANES) III reference database.^32^ Daily and weekly calibration processes were successfully followed in line with manufacturer recommendations. DXA scans were reported by an experienced and appropriately qualified DXA-reporting Associate Professor in Musculoskeletal Imaging who also had access to questionnaire responses.

### Statistical analysis

Descriptive outcome measures were collected on the following: patient demographics, prevalence of relevant clinical history, DXA outcomes (aBMD, T-Scores), FRAX fragility fracture risk estimates (10-year percentage risk), and associated estimates of aBMD and T-score from the IBEX BH software. Continuous measures are presented with sample mean and standard deviation.

Osteoporotic and non-osteoporotic subgroups were established using central DXA reports (minimum T-score of left and right NoF, and LS as per the typical clinical approach to reporting), with *P*-values testing differences between these subgroups and the entire cohort (t-test and Wilcoxon signed rank test for continuous variables, chi-squared for categorical variables). For the analysis presented here, an average was taken of the aBMD from the left and right UD ROI and similarly for the TD ROI.

The primary statistical objective was to produce a risk prediction model incorporating the IBEX BH software outputs (either the UD or the TD ROI) and commonly identifiable patient demographics (sex and age), providing a probability of clinical osteoporosis defined as central DXA T-score ≤ –2.5. The risk prediction model is a multivariate logistic regression model that is selected using forward-backward model selection using the Akaike Information Criterion (AIC). The parent model has age, sex, and T-score (either the UD or the TD ROI), plus their interaction terms. The models are analyzed using receiver operating characteristic (ROC) analysis with the area under the ROC curve (AUC) and 99% CI calculated using De Long’s method. To assess over-fitting, Leave One Out Cross Validation (CV) was also performed and the resultant AUC is also reported. If a model is overfit – the model performance on an unseen dataset is likely to be somewhere between the CV and the standard value so both are reported here. This analysis is repeated with all DXA T-scores and IBEX BH T-scores for comparison.

The secondary statistical objective was demonstrating the effectiveness of IBEX BH software in conjunction with CRFs at providing a risk prediction model for intervention, aligned with current NOGG guidance. A risk prediction model is selected that provides a probability that the NOGG guidance after FRAX (with NoF aBMD) will recommend treatment. The parent model had all CRFs and a T-score (either the UD or the TD ROI) plus the interaction terms for age, sex, and T-score. The model is selected and validated using the same methods as the probability of osteoporosis.

To compare the performance of IBEX BH as a predictor of NOGG guidance outcome using FRAX (without NoF aBMD),^5^ operating point analysis was performed. An operating point is picked such that both comparators – (a) the IBEX BH risk prediction model in the previous paragraph and (b) NOGG guidance after FRAX (without NoF aBMD) – have the same sensitivity. These are then compared with 99% CIs. The sensitivities were matched as FRAX without DXA has notably higher sensitivity than specificity so increasing specificity was considered more clinically relevant. It should be noted that this is an example operating point, and the actual point should be chosen based on the availability of DXA and other factors in an integrated care network. To assess over-fitting, CV was also performed, and the resultant specificities are reported.

Sample size estimation of 260 participants was based on power of 0.8, significance level of 0.01, prevalence of 0.33 within the sample population, an effect size of 0.1 and smallest difference of 0.05. Participants without at least one NoF DXA, one wrist DXA and one DR DXA (7/261 = 2.7%) were removed from the study. No other missing data imputation was required. Statistical analysis was performed with the statistical software package, R.^33^

## Results

Table 1 reports the continuous demographic, CRF and FRAX risk estimate summaries. Table 2 reports the categorical demographic, CRF and FRAX referral and treatment outcomes summaries. Of the 254 participants who contributed data, there was a mean age of 70.8 years (SD ± 9.0), and 142 females. About 53 participants were classified as having clinical osteoporosis.

**Table 1:** Table of continuous variables split by (i) all and (ii) osteoporosis (T-score ≤ –2.5 at either NoF or lumbar spine, as measured by DXA). The total number of patients reported in this table is *n* = 254. The mean of the group and the standard deviation (SD) of the group are reported in the first three columns. The *P*-values of a t-test and a Wilcoxon signed rank test for the difference between osteoporosis and non-osteoporotic groups are reported in the fourth and fifth columns.

	All mean (SD; *n* = 254)	Osteoporosis mean (SD; *n* = 53)	Non.osteoporosis mean (SD; *n* = 201)	*P*.value. T.test	*P*.value. Wilcoxon
Age (years)	70.839 (8.984)	74.264 (8.148)	69.935 (8.996)	0.001	0.004
Height (cm)	167.951 (9.681)	163.057 (9.145)	169.241 (9.424)	<0.001	<0.001
Weight (kg)	72.361 (14.379)	64.453 (14.344)	74.446 (13.679)	<0.001	<0.001
FRAX (without NoF aBMD) major fracture risk (10 year % risk)	12.073 (8.914)	17.896 (10.589)	10.537 (7.747)	<0.001	<0.001
FRAX (without NoF aBMD) hip fracture risk (10 year % risk)	5.134 (6.884)	8.73 (8.361)	4.186 (6.118)	<0.001	<0.001
FRAX (with NoF aBMD) major fracture risk (10 year % risk)	9.787 (7.381)	17.153 (9.903)	7.844 (5.024)	<0.001	<0.001
FRAX (with NoF aBMD) hip fracture risk (10 year % risk)	3.076 (4.764)	7.27 (7.195)	1.971 (3.059)	<0.001	<0.001

**Table 2:** Demographic factors (Osteoporosis is defined by a T-score ≤ –2.5 at either the LS or the NoF, as measured by DXA). The total number of patients reported in this table is *n* = 254. Column one reports the number of patients with that factor. Column two reports the percentage of the entire cohort with that factor. Column three reports the number with that factor in the osteoporotic group. Column four is the percentage with the factor in the osteoporotic group. Column five reports the number with that factor in the non-osteoporotic group. Column six is the percentage with the factor in the non-osteoporotic group. Column seven is the *P*-value testing whether the percentages are different between the osteoporosis and non-osteoporosis sub-groups.

	All participants (*n* = 254)	All participants %	Osteoporosis subgroup (*n* = 53)	Osteoporosis subgroup %	Non-osteoporosis subgroup (*n* = 201)	Non-osteoporosis subgroup %	*P*-value
Sex (Female)	142	55.906	39	73.585	103	51.244	0.006
Previous fracture (No)	234	92.126	43	81.132	191	95.025	0.002
Parental hip fracture (No)	218	85.827	44	83.019	174	86.567	0.662
Smoker (No)	251	98.819	51	96.226	200	99.502	0.212
Gluccocorticoids (No)	234	92.126	44	83.019	190	94.527	0.013
Rhuematoid arthritis (No)	243	95.669	50	94.34	193	96.02	0.877
Secondary osteoporosis (No)	212	83.465	41	77.358	171	85.075	0.255
High alcohol use (No)	224	88.189	49	92.453	175	87.065	0.4
NOGG treatment (No)	128	50.394	12	22.642	116	57.711	<0.001
NOGG referral (No)	208	81.89	25	47.17	183	91.045	<0.001

Table 3 presents a summary of key measurable outcomes from DXA and IBEX BH, and presents FRAX estimates and treatment recommendations. Statistically significant differences were observed between osteoporotic (*n* = 53) and non-osteoporotic (*n* = 201) subgroups for all measured sites (*P* < 0.001).

**Table 3:** DXA and IBEX BH T-score values. Osteoporosis is defined as the minimum central T-score ≤ –2.5, as measured by DXA. The number of patients contributing to each row is given in the first column. The mean of the group and the standard deviation (SD) of the group are reported in columns two to four. The *P*-values of a t-test and a Wilcoxon signed rank test for the difference between osteoporosis and non-osteoporotic groups are reported in the fifth and sixth columns. The percentage with a T-score $\le$ –2.5 is given in the seventh column. The percentage with a T-score $\ge$ –1 is given in the eighth column.

	N	Mean (SD)	Osteoporosis Mean (SD)	Non-osteoporosis Mean (SD)	P-value T-test	P-value Wilcoxon	%Tscore$\le$–2.5	%Tscore$\ge$–1
DXA spine	232	−0.564 (1.699)	−2.454 (1.078)	−0.071 (1.472)	<0.001	<0.001	14.224	54.31
DXA NoF left	245	−1.177 (1.03)	−2.342 (0.608)	−0.878 (0.896)	<0.001	<0.001	8.571	38.367
DXA NoF right	247	−1.143 (1.05)	−2.323 (0.656)	−0.828 (0.902)	<0.001	<0.001	8.097	40.486
DXA distal third left	245	−1.262 (1.391)	−2.492 (1.398)	−0.939 (1.197)	<0.001	<0.001	18.776	47.347
DXA distal third right	243	−1.212 (1.336)	−2.367 (1.415)	−0.905 (1.135)	<0.001	<0.001	18.107	46.914
DXA ultra distal left	245	−1.398 (1.952)	−3.235 (1.577)	−0.915 (1.746)	<0.001	<0.001	24.898	40.408
DXA ultra distal right	243	−1.167 (1.868)	−2.924 (1.52)	−0.701 (1.666)	<0.001	<0.001	22.634	44.444
IBEX BH distal third left	245	−1.24 (1.277)	−2.341 (1.292)	−0.951 (1.107)	<0.001	<0.001	15.102	49.796
IBEX BH distal third right	241	−1.263 (1.314)	−2.401 (1.33)	−0.965 (1.137)	<0.001	<0.001	17.427	47.303
IBEX BH ultra distal left	245	−1.387 (1.771)	−3.074 (1.614)	−0.944 (1.53)	<0.001	<0.001	26.122	42.857
IBEX BH ultra distal right	243	−1.263 (1.749)	−2.94 (1.59)	−0.817 (1.504)	<0.001	<0.001	24.28	46.091

Table 4 contains the AUCs for IBEX BH and DXA in predicting NoF T-score $\le$ –2.5 and/or LS T-score < –2.5 (i.e. a clinical diagnosis of osteoporosis for the purposes of this study). A strong performance is observed at both the UD (AUC 0.857 (0.801–0.912) 99% CI) and TD (AUC 0.815 (0.748–0.883) 99% CI) ROIs, comparable with the performance of DXA of the radius (UD AUC 0.864 (0.0.811–0.917 99%CI)) and TD (AUC 0.83 (0.0.769–0.891 99%CI)). Leave One Out CV results presented in Table 4 closely align with AUC values, suggesting low risk of the model being overfit.

**Table 4:** Area under the receiver operating characteristic curve (AUC) analysis for prediction of central osteoporosis (any NoF and/or LS T-scores $\le$ –2.5) including Ibex Bone Health (IBEX BH) and Dual Energy X-ray Absorptiometry (DXA) at the ultra-distal (UD) and distal third (TD) regions of interest. The first column reports the variables included in the parent model in the multivariate logistic regression model. The second column reports AUC with a 99% confidence interval (CI). The final column reports the cross validated (CV) AUC for the resultant model.

	AUC [99% CI]		CV AUC
Age + Sex	0.719	[0.647,0.791]	0.697
Age + Sex + DXA UD	0.864	[0.811,0.917]	0.84
Age + Sex + DXA TD	0.83	[0.769,0.891]	0.812
Age + Sex + IBEX BH UD	0.857	[0.801,0.912]	0.839
Age + Sex + IBEX BH TD	0.815	[0.748,0.883]	0.799
Age + Sex + DXA Lumbar	0.932	[0.888,0.976]	0.905
Age + Sex + DXA Pelvis	0.933	[0.895,0.970]	0.922

**Figure 3:**
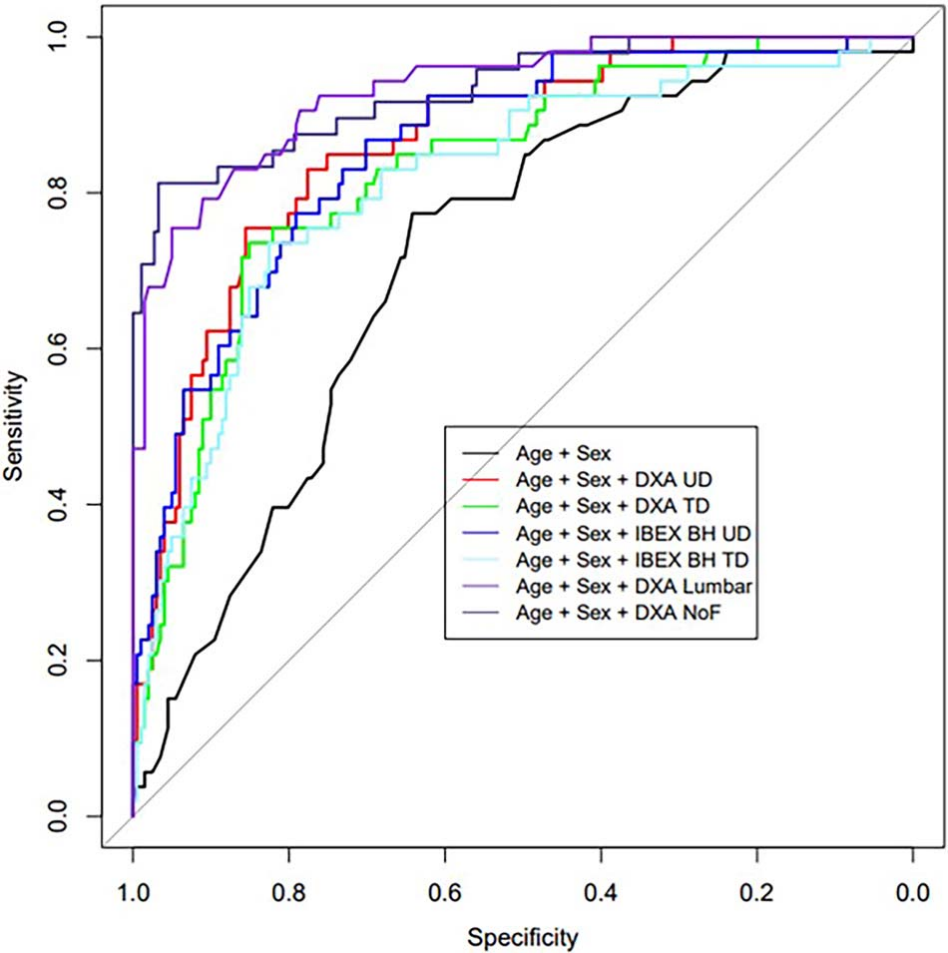
Cross validated receiver operating characteristic curves for clinical central osteoporosis (defined as the minimum central T-score ≤ –2.5, as measured by DXA) prediction (neck of femur (NoF), ultra distal (UD), distal-third (TD), IBEX BH (Ibex Bone Health), dual energy X-ray absorptiometry (DXA)).

Figure 3 shows the CV AUCs for this prediction of clinical osteoporosis. Correlation of IBEX BH T-score with T-score at the NoF is *R* = 0.66 (*P* < 0.001) and *R* = 0.63 (*P* < 0.001) for IBEX BH TD and UD ROIs, respectively.

Table 5 presents analysis of prediction of NOGG treatment recommendation by FRAX with NoF aBMD. Again IBEX BH performed strongly with AUC 0.965 (0.934–0.997 99% CI) and AUC 0.954 (0.908–1.0 99% CI) at the TD and UD sites, respectively, outperforming FRAX without NoF aBMD (AUC 0.768 (0.703–0.932 99% CI)). CV AUC values are slightly lower for both the TD (CV_AUC 0.914) and UD (CV_AUC 0.905) sites, suggesting some minor overfitting.

**Table 5:** Area under the receiver operating characteristic curve (AUC) and operating point analysis for prediction of treatment recommendation by National Osteoporosis Guideline Group (NOGG) guidelines using FRAX (with Neck of Femur (NoF) areal Bone Mineral Density (aBMD). “NOGG referral” is whether NOGG guidelines using FRAX without aBMD recommends the patient is considered low risk or sent for a Dual Energy X-ray Absorptiometry (DXA) scan, or alternatively referred straight to treatment. The second column reports the area under the AUC or specificity with a 99% confidence interval (CI). The final column reports the cross validated (CV) AUC or specificity.

	AUC (99% CI)		CV AUC
NOGG Referral	0.768	[0.703,0.832]	NA
IBEX BH TD	0.965	[0.934,0.997]	0.914
IBEX BH UD	0.954	[0.908,1]	0.905
	Specificity (99% CI)		CV Specificity
NOGG Referral	0.601	[0.51,0.688]	NA
IBEX BH TD	0.928	[0.869,0.966]	0.692
IBEX BH UD	0.894	[0.828,0.942]	0.74

**Figure 4:**
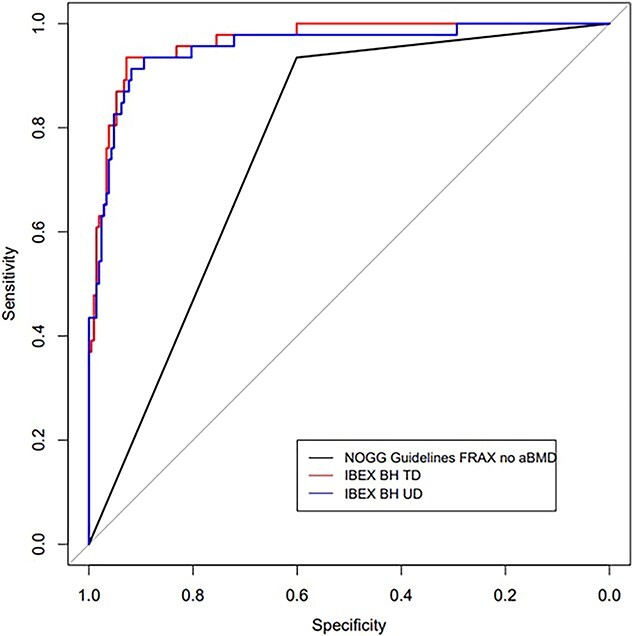
Receiver operating characteristic curves for prediction of treatment recommendation by National Osteoporosis Guideline Group (NOGG) guidelines using FRAX with neck of femur (NoF) areal bone mineral density (aBMD)). “NOGG referral” is whether NOGG guidelines using FRAX without aBMD recommends the patient is considered low risk or sent for a dual energy X-ray absorptiometry (DXA) scan, or alternatively referred straight to treatment. Curves are given for FRAX without aBMD, and IBEX Bone Health (IBEX BH) at the ultra-distal (UD) and distal-third (TD) regions of interest.

For operating point analysis, the operating point on the AUC was chosen so that the sensitivity matched FRAX (without NoF aBMD) 0.93 (99% CI [0.78, 0.99]). IBEX BH outperformed FRAX (without NoF aBMD) with specificity of 0.928 (0.869–0.966 99% CI) and 0.894 (0.828–0.942 99% CI) at TD and UD ROIs, respectively, compared with specificity of 0.601 (0.51–0.668 99% CI) for FRAX (without NoF aBMD). Figure 4 displays the cross validated AUCs for prediction of NOGG treatment recommendation by FRAX with NoF aBMD.

## Discussion

The results presented highlight the strong performance of IBEX BH for the prediction of a clinical diagnosis of osteoporosis based on DXA scanning of the hips and LS. IBEX BH also demonstrates strong predictive performance of FRAX-based treatment recommendation (when inclusive of NoF aBMD). As a software product, IBEX BH could be integrated into commercial radiographic systems and reporting pathways, potentially enabling opportunistic point of care indicators of bone health during routine wrist radiographs. These radiographs generally take only a few minutes to acquire, are simple for patients, and the use of IBEX BH requires no additional imaging or radiation burden to patients.

It is beneficial to consider the diagnostic performance of IBEX BH relative to existing comparators. Figure 4 evidences that IBEX BH resulted in an improvement when predicting the treatment results of FRAX (with NoF aBMD), over CRFs alone (FRAX without NoF BMD). Whilst IBEX BH relies on a wrist radiograph, the results suggest the potential merit of IBEX BH for the basis of treatment decisions as an alternative to FRAX with NoF aBMD, although further clinically based studies are required. Table 4 also indicates IBEX BH is a strong predictor of clinically reported osteoporosis based on LS and hip DXA scans. Results are closely aligned with the predictive ability of DXA at the wrists, which again suggest the potential of IBEX BH as a parallel or interchangeable diagnostic tool with DXA for osteoporosis decision making with further clinical development.

There remains a paradox in clinical care: while cost-effective to prevent fragility fracture, currently many healthcare centers are set primarily to respond to fragility fracture only when they occur. In the UK, FLSs have been demonstrated to reduce fragility fracture incidence and to be cost-effective.^34^^,^^35^ However, FLS inherently rely on referral or identification of those with fragility fracture, who are already at significant risk of refracture, and FLS provision is currently geographically varied within the UK. IBEX BH offers the potential for enabling an earlier diagnosis of those at risk of fragility fracture within a large target population of older patients undergoing a common radiographic examination. This may occur before or at the time of fragility fracture.

The cost-effectiveness of osteoporosis screening and benefits for patients has been considered in the wider literature.^36^^,^^37^ When considering screening specifically involving imaging, Clark et al. 2011 proposed thoracolumbar radiograph screening for those women considered at risk of vertebral fracture in a screening programme shown to increase osteoporosis medication prescription and reduction of subsequent 12-month fracture incidence.^38^ IBEX BH may fit a similar model for osteoporosis screening but instead using wrist radiographs that are easier to obtain and report.

Several alternatives to DXA have been proposed for diagnosis of osteoporosis. Quantitative ultrasound (QUS) has perhaps the largest evidence base, but meta-analysis has suggested that diagnostic accuracy is unlikely to be clinically acceptable.^39^ Other ultrasound based systems such as Bindex,^40^ based on cortical thickness at anatomical sites, and Echolight,^41^ based on Radiofrequency Echographic Multi Spectrometry (REMS) technology, demonstrate improved diagnostic accuracy over QUS relative to DXA, and comparable to IBEX BH results presented here, and also preclude the use of radiation. However, both approaches still require clinical identification and referral of those at risk to more time-consuming scan protocols, so are not opportunistic like IBEX BH, and report higher instances of missing data.^40^^,^^41^ Interestingly, research on Bindex has shown evidence that suggests novel options for earlier detection and treatment of osteoporosis can reduce reliance on DXA and be cost-effective.^42^

Other studies exist that use radiometrics at the wrist based on cortical thickness from radiographs to predict osteoporosis diagnosis and/or DXA results. The results in this study outperform those studies identified using comparable diagnostic accuracy measures.^18^^,^^20^ Differences in performance are likely to be related to the inherent approach to analysis. Whilst cortical thickness is undoubtedly linked to bone strength, there is evidence to suggest that cortical changes can occur as a result of ageing independently of aBMD.^43^ IBEX BH uses a physics-based inverse problem solving approach that solves the same fundamental problem as DXA: that a dense bone and a porous bone can exhibit equivalent intensity values in a radiograph depending on the surrounding tissue. DXA uses dual energy absorption to normalize the tissue component while IBEX BH uses a mathematical approach based on a morphological model of the body part and Monte-Carlo simulator of the X-ray system. This gives an advantage over radiometric measures as it is attempting to solve the same problem as the reference standard – rather than calculating a distinct measure and relying on its correlation to the reference standard. Furthermore, if there is independent information in radiometric measures – for example, cortical thickness – it could still be calculated from the radiograph and considered by clinicians.

### Further research

The findings of this study support further research involving the application of IBEX BH for larger patient populations and with the software applied within the clinical setting. Whilst the efficacy of the software is demonstrated here, its translation into clinical practice needs to be explored to establish a clear pathway for integration into existing clinical pathways and to demonstrate cost effectiveness and patient benefit.

In parallel to this, the encouraging results of IBEX BH at the wrist site suggest further development of analogous software for radiographs at different anatomical sites. With further research, the approach may also become valuable for specific clinical scenarios involving the wrist, such as pre-operative decision making for wrist fractures^44^ and specific conditions such as primary hyperparathyroidism where BMD loss is preferential at the distal forearm.

### Limitations

The study was carried out at a single center by a small research team. While clinical DXA protocols were followed, subjectivities potentially exist within the aspects of image analysis (such as ROI placement) and reporting within DXA. A single imaging system was used with a fixed protocol for radiographs of the distal forearm. While there is confidence from preliminary experiments in the transferability of IBEX BH to other imaging systems, larger multisite studies incorporating its use are required.

The sample population likely varies from the general population with higher prevalence of osteoporosis and bias towards females, those over 70, and having low body mass index. Furthermore, the use of a volunteer population means that it is likely fewer participants exhibited complex co-morbidities relative to the wider population of older adults. Participants did not have clinical symptoms at the time of referral and so pathology at the radius was rare. However, Tables 4 and 5 present results demonstrating that the performance of both UD and TD ROIs was comparably encouraging, meaning IBEX BH should be resilient to fracture or degenerative change affecting one ROI (most likely the UD ROI). Furthermore, Supplementary Material S1 presents data of each individual left and right ROI, with comparable results suggesting there is potential for IBEX BH to be resilient to the presence of fractures. However, further clinically-based studies are planned to improve population sampling and address these limitations.

Each participant was only measured once on the DR and DXA machines so the coefficient of variation of IBEX BH could not be inferred and compared to DXA. This is not critical for the initial intended use of the software as an opportunistic screening tool but further research to ascertain the coefficient of variation for IBEX BH is needed as it may enable its use for, for example, treatment monitoring when access to DXA is limited.

Development of a medical imaging application such as IBEX BH may involve testing a blinded dataset following product development on a separate developmental dataset. This is particularly important for data-driven components such as neural networks. However, in this study a protocol amendment was made that precluded the use of a blinded test dataset, since no data from the study were used in a training capacity for the data-driven components of the IBEX BH algorithm. While the data were used to define a mapping between IBEX BH outputs and DXA aBMD at the wrist, a linear model was used with 3 degrees of freedom fit with 488 data points and hence a blinded dataset would not have increased confidence in the algorithm sufficiently to justify the dose to these additional participants.

## Conclusion

This study demonstrates the potential of IBEX BH for the opportunistic early prediction of osteoporosis and associated fragility fracture risk, determined from a radiograph of the distal forearm. The product aligns with desirable traits of: early identification and screening initiatives; safe and low burden for patients; realistic implementation; integration with existing healthcare equipment and reporting and referral models; and a clear patient benefit to earlier identification of risk. An ongoing priority is to gather further data on the benefits of IBEX BH within a clinical setting, and the development of IBEX BH for alternative common radiographic examination sites.

## Supplementary Material

supp_materials1_ziae020

## Data Availability

The data that support the findings of this study are available from the corresponding author upon reasonable request.
