# Supplementary material for: Development of an opportunistic diagnostic prediction algorithm for osteoporosis and fragility fracture risk estimates from forearm radiographs (The OFFER1 Study)
Source: JBMR Plus. 2024 Mar 15;8(4):ziae020. doi: 10.1093/jbmrpl/ziae020 (PMC10945724; doi:10.1093/jbmrpl/ziae020)
Supplement: supp_materials1_ziae020 [file supp_materials1_ziae020.docx]

**Supplementary Material 1.**

Supplementary table 1: Area under the receiver operating characteristic curve (AUC) analysis for prediction of central osteoporosis (any NoF and/or LS T-scores $\leq$ -2.5) including Ibex Bone Health (IBEX BH at the ultra-distal (UD) and distal third (TD) regions of interest. The first column reports the variables included in the parent model in the multivariate logistic regression model. The second column reports AUC with a 99% confidence interval (CI). The final column reports the cross validated (CV) AUC for the resultant model.

|  | AUC [99% CI] | | CV AUC |
| --- | --- | --- | --- |
| Age + Sex + IBEX BH UD Left | 0.853 | [0.792,0.913] | 0.84 |
| Age + Sex + IBEX BH UD Right | 0.85 | [0.783,0.907] | 0.825 |
| Age + Sex + IBEX BH TD Left | 0.813 | [0.743,0.882] | 0.795 |
| Age + Sex + IBEX BH TD Right | 0.808 | [0.738,0.879] | 0.792 |
| Age + Sex + IBEX BH UD Minimum | 0.854 | [0.798,0.910] | 0.841 |
| Age + Sex + IBEX BH TD Minimum | 0.807 | [0.737,0.876] | 0.785 |

Supplementary Table 2: Area under the receiver operating characteristic curve (AUC) and operating point analysis for prediction of treatment recommendation by National Osteoporosis Guideline Group (NOGG) guidelines using FRAX (with Neck of Femur (NoF) areal Bone Mineral Density (aBMD). The second column reports the area under the AUC with a 99% confidence interval (CI). The final column reports the cross validated (CV) AUC.

|  | AUC (99% CI) | | CV AUC |
| --- | --- | --- | --- |
| FRAX (No NoF aBMD) | 0.883 | [0.811,0.955] | NA |
| IBEX BH UD Left | 0.954 | [0.907,1] | 0.905 |
| IBEX BH TD Right | 0.951 | [0.902,1] | 0.900 |
| IBEX BH UD Left | 0.951 | [0.907,0.995] | 0.901 |
| IBEX BH TD Right | 0.971 | [0.943,1] | 0.921 |


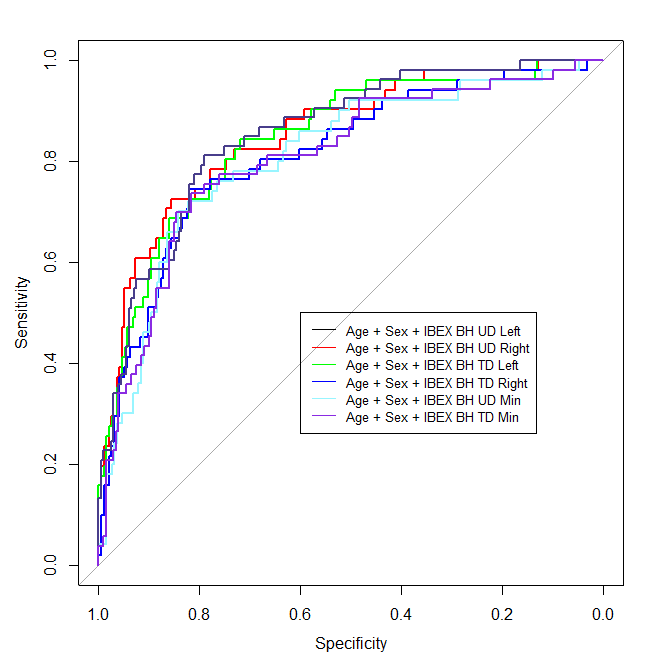


Supplementary Figure 1: Receiver operating characteristic curves for clinical central osteoporosis (defined as the minimum central T-score ≤ -2.5, as measured by DXA) prediction (Neck of Femur (NoF), ultra distal (UD), distal-third (TD), IBEX BH (Ibex Bone Health), Dual Energy X-ray Absorptiometry (DXA)).

Supplementary Figure 2: Receiver operating characteristic curves for prediction of treatment recommendation by National Osteoporosis Guideline Group (NOGG) guidelines using FRAX with Neck of Femur (NoF) areal Bone Mineral Density (aBMD)). “NOGG referral” is whether NOGG guidelines using FRAX without aBMD recommends the patient is considered low risk or sent for a Dual Energy X-ray Absorptiometry (DXA) scan, or alternatively referred straight to treatment. Frax Major is the 10-year probability of major osteoporotic fracture from FRAX without NOF aBMD.
